# Quality of care during childbirth in Tanzania: identification of areas that need improvement

**DOI:** 10.1186/s12978-018-0463-1

**Published:** 2018-01-27

**Authors:** Andrea Solnes Miltenburg, Richard Forget Kiritta, Tarek Meguid, Johanne Sundby

**Affiliations:** 1 Institute of Health and Society, Department of Community Medicine and Global Health, Faculty of Medicine, University of Oslo, Oslo, Norway; 2 Department of Obstetrics and Gynaecology, Sekotoure Regional Referral Hospital, Mwanza, Mwanza Region Tanzania; 3 Department of Obstetrics & Gynaecology, Mnazi Mmoja Hospital, Zanzibar, Tanzania

**Keywords:** Maternal Health, Quality of Care, Participant Observation, Partograph, Active Management of Third Stage of Labour

## Abstract

**Background:**

Making use of good, evidence based routines, for management of normal childbirth is essential to ensure quality of care and prevent, identify and manage complications if they occur. Two essential routine care interventions as defined by the World Health Organization are the use of the Partograph and Active Management of the Third Stage of Labour. Both interventions have been evaluated for their ability to assist health providers to detect and deal with complications. There is however little research about the quality of such interventions for routine care. Qualitative studies can help to understand how such complex interventions are implemented. This paper reports on findings from an observation study on maternity wards in Tanzania.

**Methods:**

The study took place in the Lake Zone in Tanzania. Between 2014 and 2016 the first author observed and participated in the care for women on maternity wards in four rural and semi-urban health facilities. The data is a result of approximately 1300 hours of observations, systematically recorded primarily in observation notes and notes of informal conversations with health providers, women and their families. Detailed description of care processes were analysed using an ethnographic analysis approach focused on the sequential relationship of the ‘stages of labour’. Themes were identified through identification of recurrent patterns.

**Results:**

Three themes were identified: 1) Women’s movement between rooms during birth, 2) health providers’ assumptions and hope for a ‘normal’ birth, 3) fear of poor outcomes that stimulates intervention during birth. Women move between different rooms during childbirth which influences the care they receive. Few women were monitored during their first stage of labour. Routine birth monitoring appeared absent due to health providers ’assumptions and hope for good outcomes. This was rooted in a general belief that most women eventually give birth without problems and the partograph did not correspond with health providers’ experience of the birth process. Contextual circumstances also limited health worker ability to act in case of complications. At the same time, fear for being held personally responsible for outcomes triggered active intervention in second stage of labour, even if there was no indication to intervene.

**Conclusions:**

Insufficient monitoring leads to poor preparedness of health providers both for normal birth and in case of complications. As a result both underuse and overuse of interventions contribute to poor quality of care. Risk and complication management have for many years been prioritized at the expense of routine care for all women. Complex evaluations are needed to understand the current implementation gaps and find ways for improving quality of care for all women.

**Electronic supplementary material:**

The online version of this article (10.1186/s12978-018-0463-1) contains supplementary material, which is available to authorized users.

## Plain English summary

Good routines for management of normal childbirth are essential to ensure quality of care and timely actions in case of complications. There is however little research about the quality of routine care. This paper reports on findings from the maternity ward, from an observation study in Tanzania. The study took place in the Lake Zone in Tanzania. Between 2014 and 2016 the first author observed and participated in the care for women on maternity wards in four rural and semi-urban health facilities. The data is a result of approximately 1300 hours of observations. Our findings show that facility infrastructure requires women to move between different rooms during active childbirth. This influences the care they receive. We observed few women were monitored during birth. This is partly because health workers have an assumptions and hope for good outcomes. At the same time health workers fear for being held personally responsible for outcomes. This triggers the use of interventions to speed up birth even if there was no indication to intervene. Insufficient monitoring leads to poor preparedness of health providers both for normal birth and in case of complications. Risk and complication management have for many years been prioritized at the expense of routine care for all women. To understand the current gaps between what care is desired and what happens in reality complex evaluations are needed to find ways for improving quality of care for all women.

## Background

During the past two decades the global maternal mortality ratio (MMR) has nearly halved. Although this is reason for optimism, many countries in sub Sahara Africa did not achieve the Millennium Development Goal 5 target of 75% MMR reduction [1]. Nevertheless, in many of these countries there was substantial increase in coverage of facility births [2]. Persisting high mortality and morbidity figures despite increase in service utilization, is thought to be due to a gap in the quality of care [2, 3]. Quality of care is insufficient due to poor facility capacity to provide timely emergency obstetric care (EmOC) and limited health provider knowledge to prevent, identify and manage complications [4]. Additionally, the push for increased numbers of facility births in countries with weak health systems also threatens the quality of routine care for uncomplicated births.

High quality of childbirth care includes provision of both routine care and emergency care. The majority of women giving birth in the health facility only require routine care. One reason for coming to a facility is to be near interventions provided by competent personnel if a complication occurs. Lack of good quality routine care may lead to more complications or late detection of these. However limited systematic research is available on the quality of routine care in low-middle income countries [4]. Quality of care assessments for management of complications are guided by the EmOC signal functions but similar signal functions for routine care are not well defined [4–6]. The WHO quality standard for childbirth care [7] suggests a selected number of process measures for quality assessment of routine monitoring during childbirth covering two essential interventions: 1) initiation and use of the partograph for monitoring and management of first and second stage of labour and 2) active management of the third stage of labour (AMTSL). Both interventions are intended to allow for controlled monitoring of the natural processes of birth, early identification of complications and preparedness of potential interventions when things deviate [8].

Use of the partograph and AMTSL are complex interventions because they contain a variety of interacting components and rely on good clinical skills [9]. Both interventions have been evaluated for their ability to help health providers detect and deal with complications. Little attention however has been paid to such intervention as part of routine care [4] beyond assessment of coverage of selected elements (e.g administration of oxytocin). It is increasingly acknowledged that there is a gap between global strategies, coverage of services and what actually happens when women reach health facilities to give birth [10]. Measurement of coverage of services and outcomes of care, although relevant, do not inform us on how care is provided and under which circumstances. Nor does it inform us about the health workers evaluation of clinical findings and decision making process. Qualitative studies are therefore important to increase our understanding of how such care processes take place and what this tells us about the quality of interventions such as the partograph and AMTSL for routine care [11]. This paper reports on findings from an observation study in Tanzania. Through description and holistic analysis of partograph use and AMTSL we intend to provide an understanding of the quality of routine care for uncomplicated childbirth in an African setting.

## Methods

### Setting

The study took place in the Lake Zone in Tanzania. Although Tanzania has made significant progress between 1999 and 2015 in increasing facility births (from 47% to 63%) the maternal mortality ratio has remained the same (from 578 in 2000 with CI 466-690 to 556 in 2015 with CI 446-666) [12]. Other sources suggest there has been substantial progress (MMR of 398 in 2015, CI 281-570) [13] but current reduction rates and wide confidence intervals suggest it remains unlikely Tanzania will reach the 2030 target MMR of 140 maternal deaths per 100.000 live births [14]. Maternity care in Tanzania is organized along a referral pyramid with dispensaries being the first formal health unit at primary level, followed by health centres and district hospitals [15]*.* All facility levels are expected to provide routine care for pregnant women and basic EmOC. District hospitals are expected to provide Comprehensive EmOC including availability of blood transfusion and caesarean sections [16].

### Observer and Observations

Over the course of 25 months between 2014 and 2016 the first author [ASM], a medical doctor, observed and participated in the care for women on maternity wards in four health facilities. ASM speaks Kiswahili and lived in Tanzania for the past three years engaging with peoples lives in both urban and rural settings. For 6 months, ASM taught nursing and medical students on a weekly basis while volunteering alongside midwives and doctors on the maternity ward in one urban district hospital. This was followed by 8 months of volunteering full time as a medical doctor at a semi-urban faith-based health centre, before entering a systematic PhD program. As the research role evolved the final 11 months more focused observations were performed in a second rural district hospital and rural government health centre where the researcher had no clinical responsibilities. During these final months a research assistant (a nurse) also provided observations at the district hospital. As she was less able to intervene and participate this helped to increase understanding of routine care processes. For practical reasons observations only took place during the daytime and evenings. The research role of observing versus participating often fluctuated and shifted depending on the situation.

Being a medical doctor, ASM was naturally engaged in the defences of best practices in obstetric care from a technological point of view, following essential guidelines as promoted by the WHO. As a result of this, beyond participating in care provision, there were situations where ASM would intervene in normal care. For example if the situation was interpreted to potentially endanger the woman (either physically or psychologically) or cause unnecessary harm. Intervening in normal care included active discussion with providers about the course of action, give solicited or unsolicited advice or take action.

Taking action would always be in agreement with the women, the health providers or their seniors and only if the researcher felt capable to do so. For the final 11 month of observations the researcher had no authority to intervene independently. This was clear to the staff because observations were not on a daily basis and the researcher never established a formal role in these institutions. On some occasions the researcher faced difficult decision whether to intervene in the process of care or not. Situations occurred where women were prepared for a caesarean section without proper indication or where women received harmful treatment or practices, which went against most standards of care. When the researcher intervened this sometimes resulted in clashes or arguments with some of the staff. When the researcher did not intervene, and remained in the observer role, feelings of guilt for allowing things to evolve gave internal conflict. If the researcher intervened this was documented in the observation reports and taken into account for analysis.

### Data collection

The data is a result of approximately 1300 hours of participant observation and consist of systematically recorded observation notes, notes of informal conversations with health providers, women and their families. In addition we reviewed clinical notes, handover books, staff schedules, reporting books and clinic cards while being present at the facilities. Observations mainly focused on the perspective of the health provider. During observations, activities and timing of care provision for individual women were documented using key words if the situation allowed for it. This usually started with description of the situation on the maternity wards after arrival. Researchers selected women that arrived at the nursing station or that were already admitted with what was expected to be an uncomplicated birth. Where possible individual women were followed until they had given birth. In additional other remarkable situations were noted, this could include situations where women developed complications. Although focus for our observations was uncomplicated births, it was important to document other happenings as they influenced and determined health worker’s other activities. Full observation reports were written after each observation day. To the best of the researchers ability details of the day were documented. In addition personal reflections including feelings and interpretation of the activities were written down in the same reports.

### Data analysis

Preliminary analysis of findings took place throughout the observation period. As a reference to standards of care we made use of a modification of the Maternal and Newborn Quality of Care Survey Labor and Delivery Observation Checklist developed by the Maternal and Child Health Integrated Program (MCHIP) [17]. The modified checklist is provided as Additional file 1. Detailed analysis was done after completion of the data collection using an ethnographic analysis approach comprising of four subsequent techniques: domain-, taxonomic-, componential- and theme analyses [18]. Domain analysis helped to meaningfully describe observations. For example one domain that we identified was ‘the admission process’ and all activities that these include (e.g. review of antenatal record, history taking, physical examination). For this paper domain analysis focused on identifying the ‘stages of labour’^1^ (see Table 1). Our reference point and terms used included the three stages of labour, around which standards of care have been organized in both international and local protocols with focus on normal, uncomplicated, childbirth.

**Table 1:** Stages of Labour [8]

*Latent phase*
Presence of contraction which can both be regular and irregular, cervical dilatation < 4 cm
*Active phase*
*First stage*: Presence of frequent and regular contractions, cervical dilatation > 4 cm
*Second stage*: Full cervical dilatation and urge to push, birth of the baby
*Third stage*: After birth of the baby, placental separation and expulsion

Taxonomy analysis resulted in identification of sub-domains within the different stages of labour (e.g movements between stages, locations of service provision, actions performed, consequences of clinical findings etc). This helped to identify how procedures and actions were organized and understand behaviour of women and health providers in the different stages and how they related to each other. Subsequently compound analysis helped to identify contrasting dimensions and its attributes (e.g. characteristics of woman and health providers, expectations of services, actual observed practices for monitoring and actions taken in case of deviations across the stages of labour). Themes were identified through identification of recurrent patterns in the previous steps.

## Results

During analysis the following themes were identified: 1) Women’s movement between rooms during birth, 2) health providers’ assumptions and hope for a ‘normal’ birth, 3) fear of poor outcomes that stimulates intervention during birth.

### Moving between rooms

Women accessed at least two rooms during childbirth at the health facility: the waiting room and/or the birth room (see Table 2).

*In the maternity ward, women who are in latent phase or early stages of childbirth are admitted to the waiting room or antenatal room. In practice, this includes all women who are in the first stage of labour. There are 8 beds in the waiting room and women often have to share a bed when it is crowded. [...] When approaching the second stage of labour, women need to shift to the birth room. Women need to walk approximately 15-20 meters to get to this room, carrying their own belonging and supplies. On their way to this room women pass the nursing station, which is located adjacent to the birth room. The birth room has six delivery beds, three on each side, although one of the beds is mainly used for supplies and materials. Rarely all beds are occupied. (Observation note district hospital)*

**Table 2:** Matrix of stages of labour in waiting area and birth room

Stage	Latent	Early first	Late first	Early second		Late second	Third
Location	Waiting area			Birth room			
Birth expected	Birth not imminent			Birth imminent			NA
Labour	Not in labour	Beginning in labour		In labour			After birth
Cervix	Tip of finger, closed,<4 cm	3-7cm	7or 8cm	8or9cm	Fully dilated head high/far	Fully dilated head close	NA
Time allowed	Days	Up to 1 day	Up to 6 hours	Up to 4 hours	1-2 hours	1 hour	5-15min
Nursing proximity	Far			Hearing distance		Close by	
Monitoring frequency	None	4hourly, upon shift change, doctors round, upon request		Frequent		None	
Monitoring activity	None	Cervical dilatation, foetal heart rate		Cervical dilatation		None	
Care seeking	Woman needs to approach the nurse by walking to the nursing station			Nurse will approach the woman, woman can ask for help when nurses are in hearing distance or on the labour ward			
Visitors/birth companion	Allowed on set times inside but women are able to go outside as they please			Not Allowed, only when instructed by nursing staff to bring supplies and materials			
Mobility	Women are able to walk around freely, urinate at the toilet, can position herself in any way but has to lie flat on her back during examination			Women are confined to the labour bed, urinate in a basin next to the labour bed which is brought by the woman herself, has to position herself lying flat on her back for pushing or other nursing activities			
Food and drink	Is allowed to eat and drink as she pleases			Is only allowed tea when this is instructed by the staff			

The admission process at the nursing station was the start of the care process. Examination was part of this process and took place in the birth room. Most of the time, it included assessments of the fundal height, fetal heart rate and vaginal examination to determine the cervical dilatation. Women arriving before the second stage of labour were considered not to be ‘in labour’ and they were placed in the waiting area together with women who were not in active phase of labour (for stages of labour see Table 1). When the beds in the birth room were not occupied, nurses would say ‘it is not busy’ or ‘we don’t have anyone in labour’ while several women could be in active stage of labour in the waiting room. Women were required to walk from the waiting area to the birth room for physical examinations, complete certain stadia of birth within an expected timeframe (e.g cervical dilatation of 1 cm per hour) and adhere to the rules of the health facility (e.g. ensure availability of necessary supplies, wear appropriate clothing) for each step in the process. Although most births fit well into this local flow of care provision, some women had a faster birth than expected. For example Mrs P (18 years, primigravida).

*Mrs P came to the hospital at 10.40am. At 11 am the nurse examines her abdomen and checks how far her cervix has dilated. She then informs Mrs P that she has to try and walk around, drink tea and can come back later: ‘After four hours we measure you again’. Mrs P goes to the waiting area and the nurse documents that she has a cervical dilatation of 7cm on the admission form and the partograph. An hour later, another nurse brings Mrs P back to the nursing station, saying: ‘This mama was pushing above her basin’. The nurse informs Mrs P it is not yet time. She should drink her tea and come back when she feels strong pushing urge. Mrs P quietly walks back to the waiting area. After 5 minutes, another pregnant woman comes to inform the nurses someone is pushing in the waiting area. Not long after cries of a baby can be heard and Mrs P has given birth to her daughter in the waiting area, assisted by her own mother. (Observation notes District Hospital)*

### ‘Whatever will be, will be’

After admission women’s antenatal card and files including a partograph remained at the nursing station. The type of partograph used at the health facilities was the composite partograph and was integrated in the admission form used at the maternity ward. Partographs were often not filled in immediately after admission, even though women where in active phase of labour. There was usually no organized filing system and from the admission files it was not clear which women required monitoring or were expected to give birth the coming hours. Sometimes women were admitted for days without any notes of observations until they eventually presented themselves at the nursing station in second stage of labour. The following case of Mrs S (32 years, gravida 6, para 5) illustrates poor monitoring and insufficient use of the partograph:

*At 11am, the nurse says they are having a prolonged labour case, which they are thinking of referring. When we go to review the case of Mrs S, it becomes clear no partograph was filled in so the nurse decides to fill in her findings of this morning. Mrs S, who has given birth 5 times before with only two living children, informs us she was checked twice, upon arrival at 10pm yesterday evening and early morning at 6am. Some scattered notes show us her cervix was 5 cm dilated on arrival. There is no documentation of the examination of 6am, but the nurse checked Mrs S at 9.30am after the ward rounds and found her cervix was 6 cm dilated. (Observation notes Health Centre)*

Routine ‘checks’ and monitoring of women in the active stage occurred primarily during nurses’ shifts changes (around 7am, 1pm and 7pm) and if there was a doctors ward round (between 9 and 10am). Nurses would collect all admission papers and pass by the different rooms on the maternity wards. If vaginal examination was needed women were asked to come to the nursing station one by one after the ward round. The time intervals between such routines during daytime were relatively short which allowed for somewhat regular monitoring. However, during the evening and night shifts many women like Mrs S were often left unattended and unnoticed until morning hours. Sometimes women presented themselves at the nursing station requesting to be examined

If the partograph was used, nurses appeared to prioritize some parts of the partograph, while leaving out others. If examination took place for the purpose of monitoring, this primarily included routine assessment of cervical dilatation. Less invasive assessments such as blood pressure measurement, assessment of fetal heart rate, number of contractions per 10 minute, pulse rate or temperature were rarely measured nor discussed in relation to the birth progress or well-being of the mother. Blood pressures were sometimes documented based on the findings during the last clinic visit, because the blood pressure machine was not readily available and a temperature of 37.5 C was often written assuming the woman had no fever. Assessment findings were not always recorded on the partograph, but rather in separate notes. Some assessments were filled in through ‘guessing’ rather than actual measuring.

*While assisting the nurse with documentation on the partograph, I asked her how much the fetal heart rate was. She listened for a few seconds with the fetoscope and said ‘136’. I wondered how she calculated this so quickly. [Observation notes district hospital]*

Usually nurses expressed an expectation that, even though based on the partograph the firsts stage appeared to take too long, most women would eventually give birth without problems and with mostly positive outcomes. If women seemed to approach the action line on the partograph nurses would sometimes dismiss previous documented cervical dilatation or make changes to what was filled in on the partograph before and rather allow women more time to progress without intervention. Sometimes nurses preferred to avoid documentation on the partograph or did not actively monitor women unless women themselves called the nurses for assistance. This lack of monitoring could result in staff being unprepared for in-facility ‘sudden events’. Women who had been admitted for days could appear with ‘sudden’ onset of complications, give birth ‘suddenly’ on their own in the waiting area or ‘suddenly’ appear in second stage at the birth room with staff being unprepared, for example Mrs D (19 years, primigravida).

*After finishing at the ANC clinic, at 1pm, a nurse rushes to the birth room where she quickly puts on some gloves. A young girl, Mrs D, is lying on the bed in second stage of labour. The nurse says: ‘This girl was admitted to the ward since yesterday, now suddenly she comes in second stage’.[…] Her file states that Mrs D was examined this morning and that her cervix was 2 cm dilated. […] Shortly after, she gives birth to a 3kg baby girl. (Observation notes Health Center)*

Nurses were also hesitant to start the partograph at early first stage of labour, saying this would result in ‘too many women reaching the action line’. The action line would indicate ‘poor progress’ requiring immediate action, which was not always easy to manage in their setting. The decision that birth was not progressing resulted in a range of consequences for both families and health providers in the example of Mrs. S:

*When we examined Mrs. S at 11am she had mild contractions but a cervical dilatation of 8 cm with the head high and membranes intact. The fetal heart rate was fine. There was hesitation to rupture the membranes because of the long distance to the district hospital in case of problems after rupturing. […] The doctor- in charge, who was on his way to the hospital, instructed the nurse to rupture the membranes and to give Mrs. S 500ml IV fluids. […] There was slight meconium stained fluid but a good fetal heart rate of 140 beats per minute [bpm]. At 12.30 Mrs S was still 8 cm and not showing signs of progress and the nurse suggested to refer her for further management. But lack of available ambulance would require the family to make their own arrangements.[…] The doctor in-charge was still on his way and informed us he would come to perform a caesarean section. […] The family was instructed to buy a catheter and the nurse asked the laboratory technician to perform hemoglobin measurement. The doctor arrived at 2pm. Meanwhile the contractions of Mrs S had increased, although she had progressed, there were signs of obstruction. Family members were asked to bring supplies and water for cleaning the operating theatre at 3pm. Finally at 4pm Mrs S delivered a baby boy by caesarean section. (Observation notes Health Center)*

In the example of Mrs S several delays occurred after poor progress was diagnosed. The nurses, and the researcher, experienced they had little influence on these delays. The researcher had suggested to drive Mrs S to the district hospital in her private car. Although both the nurses and Mrs S where happy with this option the medical doctor refused this saying he would arrive soon to assess the situation. When he finally arrived, family members where instructed to make necessary arrangements before the caesarean section was possible, resulting in more delay. Similar delays were also observed at the district hospital. One afternoon nurses adequately and timely called for supervision for a case of obstructed labour. It took an entire night before the woman was taken to the operating theatre the next morning.

### Active management out of fear of poor outcomes

After women arrived in the birth room we observed nurses appeared to have a sense of urgency to intervene and make sure women gave birth as quickly as possible. Failure to complete a stadium, for example poor descend of the head within the expected time, or failure according to expected social norms, such as screaming out of pain and being perceived demanding, was considered a deviation of ‘normal’ and required intervention and active management. If women did not give birth soon enough after arriving to the birth room nurses would fear poor progress would lead to poor fetal outcome and therefore required active management. For example Mrs A (21 years, gravida 3, para 0), who was lying in the birth room with what was perceived to be poor progress because she had been in the birth room since early morning. The following events occurred:

*10.25: Nurse gives 500ml IV fluids and a urine catheter. She struggles with the catheter and it seems painful for Mrs A.*

*10.36: Doctor comes in and asks what size the catheter is and leaves right after.*

*10.39: Nurse looks at the partograph and discusses this with the doctor who agrees to give her some more time.*

*11.50: Gives second bag of IV fluids. There is still no urine in the urine bag. Doctor also comes and looks at her but does nothing.*

*12.10: Mrs A says she feels to push. The nurse comes and talks to her.*

*12.30: Doctor orders to prepare for a caesarean section and orders to check the Hb.*

*13.10: Researcher intervenes: it is documented her membranes are still intact; the nurse decides to check this and does a vaginal examination of Mrs A. She finds Mrs A has intact membranes and only 4cm cervical dilatation. She discusses with colleagues to find out who documented she had 6cm several hours ago.*

*13.20: Discussions with the doctor who also performs a vaginal examination. He instructs removal of the catheter and IV line and tells Mrs A to go to the waiting area and come back for another check after 4 hours. ‘She is not in labour’ he says.*

*13.30: The nurse checks the FHR and Mrs A collects her things to go to the waiting area.*

*(Observation notes District Hospital)*

Nurses would quickly provide women with IV fluids, even though women were able to drink, have catheters inserted, while women were able to urinate spontaneously, give oxytocin, while contractions were sufficient or give episiotomies or fundus expression while there was no information regarding the fetal condition due to lack of assessment of the fetal heart rate.

The following case of Mrs D describes active management of the third stage of labour:

*She [the nurse] felt the top of the uterus and then started pulling the cord, the cord and placenta followed smoothly without problems. She dropped the placenta into the bunch of ‘kitenge [local fabric]’ lying on the bed without looking at it. She looked at the perineum, while spreading the labia with one finger. ‘No tear’, she said. Then she started pushing heavily on the fundus of the uterus to push blood clots out of the vagina: pushing deep and massaging. Meanwhile some blood was leaking on the floor, so she told Mrs D to hold the plastic sheet up to avoid it from dripping down. The nurse looked for some fabric to clean the blood away and took the basin towards her with her foot. Then she took of one glove on her right hand as she was wearing another one underneath. She then went inside the vagina with the same hand to take out some more remaining clots. She did this several times while at the same time pushing on the stomach with her left hand for about 5 minutes long while Mrs D was moaning of pain. The nurse said: ‘Look how much blood, so much blood’. (Observation notes Health Center)*

The example above was hardly an isolated event and was observed both at health center level and at the district hospitals. Provision of oxytocin and controlled cord traction were usually provided for all women. Although posters of recent training on prevention of post-partum hemorrhage were hanging on the walls of the wards, it was not accepted that expulsion of the placenta could take up to 30 min after birth, the placenta was rarely checked for completeness and uterine tone was assumed not to be sufficient, even if the uterus was well contracted. Instead, uterine massage and manual expression to empty the uterus for blood clots was done on many occasions.

Despite frequent absence of monitoring and unclear indications for interventions, outcomes of births were mostly good. Maternal death remained a rare event, and it seemed generally acknowledged to be a result of multifactorial causes. Nevertheless, in particular neonatal deaths and stillbirths occurred relatively frequently. Renewed focus on these deaths, both by the ministry and media^2^ increased health providers’ fear to be personally held accountable for such outcomes. These fears became mostly visible in decisions made around documentation to avoid potential blame.

*‘Don’t teach the students that [to fill in the partograph]. This will be used in court. If the baby dies you will not be blamed but I don’t want to loose my job. I can loose my job for someone else's mistake’ (Reflection notes after teaching students on the maternity ward)*

We observed partographs were often completed after birth, visualizing regular monitoring of fetal heart rate every 30 minutes and linear increase of severity of contractions combined with linear increase of cervical dilatation, even if this was not the case. For example, after poor progress was diagnosed for Mrs S, the nurse retrospectively filled in all FHR measurements on the partograph of the night before:

[Researcher]: ‘Why are you filling this in now?’

[Nurse] : ‘It needs to be completed’.

[Researcher]: ‘But you did not check this right?’

[Nurse]: ‘No, but the nurse of the night was there’

[Reseacher]: ‘Yes but did he actually check it [the FHR]?’

[Nurse]: ‘No, probably not’.

(Conversation extracted from observation notes Health Centre)

Review of reporting books showed Apgar scores where nearly always reported to be 9 after 1 minute and 10 after 5 minutes. We also observed documentation of more positive Apgar scores than what the score would be in reality. On one occasion we reviewed the case of a neonatal death at a health center, which had occurred during the night shift. The Apgar score had been documented to be 10 after 5 minutes. The nurse on duty decided to change this to 6 after 5 minutes because a score of 10 had seemed unlikely because the baby had passed away. The majority of cases that may have been a neonatal death were recorded as fresh stillbirths. Stillbirth cases where a fetal heart rate had been documented on admission were sometimes revised to indicate death had likely occurred before arrival at the facility.

## Discussion

In this study we present a range of examples of what can be considered suboptimal maternal health care. Interventions and activities that are intended to support health providers to monitor the natural processes of birth are not used accordingly. For example the partograph seems to be used more as a documentation tool than a decision-making tool. Often this is the result of a non-conducive environment, which hinder their effective use. In addition a culture of ‘blame and shame’ creates unfavourable working conditions where individuals perceive to be held accountable for system failures. Our findings demonstrate how this negatively influences care provision. It must be recognized that these observations, findings and shortcomings are not intended to point fingers at the individual providers or facility managers. Facility or care providers names or titles are not included in the paper and most of them work as good as possible in the setting in which they are operating. It is however a reality that much of the routine care provided is sub-optimal and is also taught in sub optimal institutions. Rather than viewing this as individual mistakes, it needs to be acknowledged that the underlying reasons for sub optimal care provision are much more complex, and have roots in poor economic conditions, work organization shortcomings, lack of supervision and poor priority setting at the political level.

The observations are limited to few health facilities in the Lake Zone region, but the setting here is not fundamentally different from any other setting in SSA.^3^ The underlying issues with regards to health system challenges, poor availability of resources and increasing pressure to improve maternal and neonatal outcomes are similar to other low-resource settings. We believe our findings are relevant beyond our setting in Tanzania, illustrating the importance of qualitative methods to understand complex care processes. Being a doctor and an observer at the same time has both benefits and drawbacks. As a doctor, the internalized idea about standards of care gave an urge to intervene and this perspective also influenced the data collected and how it was analysed. It also limited the ability to meticulously document complete care processes for individual women. Nevertheless joining local health staff proved a unique opportunity for detailed informed observations of provision of care.

Through our analysis we identified health providers are confronted with contrasting drives for care provision during the birthing process. In circumstances such as in these Tanzanian health facilities, health providers appear to balance the perception of not being able to influence the process of care with the fear that they will be held responsible for the outcome. On the one hand care provision during the first stage of labour can appear meaningless resulting in inaction. On the other hand, during second and third stage of labour actions determine outcomes, stimulating the use of interventions. In the following section we will argue that the tandem of underuse and overuse of care during childbirth occurs due to a failing technocratic model of care and decades long prioritization of risk and complication management at the expense of routine care for all women.

### Failure of the technocratic model in unpredictable settings

Dividing the birth process in stages and monitoring of individual women with the partograph is part of the development of the technocratic model of childbirth [1]. Applying this model, with increased use of technology, routines and protocols for monitoring and management of childbirth has improved outcomes but is also criticized for viewing birth as a mechanical process [19]. The partograph, developed based on the Friedman curve, is based on the expectation that birth occurs in a certain time frame [20, 21]. Approaching birth as a linear process can result in an “assembly line” of care provision, not paying attention to individual variations in the birth process [22], as was observed in this study. Although the partograph is thought to be too stringent [22, 23] and evidence of positive impact on outcomes remains limited [24] its use is still advocated in settings with high maternal mortality and morbidity [7]. The partograph functions as an ‘early warning system’ [21, 25], and intends to assist clinical management of childbirth and timely identify poor progress [26]. Nevertheless despite its widespread promotion for the past decades many studies have shown partographs are not implemented well: they are often not used at all, filled in after birth, or filled in incomplete [27–32].

We found that the hesitancy of health providers to initiate the partograph lies on the one hand in the assumption that most women will have an uncomplicated birth despite crossing the action line on the partograph and on the other hand that care processes are perceived to be out of their control. The partograph implicitly stimulates the ‘hunt for abnormality’ and generates an individual responsibility of childbirth management where health providers can be held accountable for the outcome [33]. However, when birth is in transition to deviate from what is accepted as the norm, care provision can become highly unpredictable as a result of ineffective referral systems, unavailable supervision staff and lack of timely available necessary supplies and materials [4, 30, 34, 35]. Use of the partograph is unlikely to improve outcomes in an ineffective maternity care system which still causes outcomes of childbirth to be a ‘product of chance’ [25, 33, 36]. Health providers seem to ‘buy time’ for both women and themselves, to allow birth to progress without interventions [37]. It is not simply insufficient use of the partograph, which results in poor monitoring. Monitoring is poor because nurses perceive they lack the ability to provide meaningful care to women in the first stage of labour. For this reason implementation of the use of the partograph in such settings seems largely unsuccessful.

### Treatment in the absence of risk

In the absence of monitoring, which extends into the final stages of labour, active management takes over out of fear for individual consequences in the case of poor outcomes [38]. Low resource settings are usually associated with insufficient availability of interventions, nevertheless, both over medicalization and overuse of interventions form an increasing threat to quality of care, also in Tanzania [39–41]. Some authors report on a high incidence of unnecessary caesarean sections in some Tanzanian health facilities [42–44], partly explained by poor implementation of guidelines [42] and increasingly defensive clinical decision-making [38]. For health workers working in settings of uncertainty, providing as much intervention as possible can give a sense of control, hoping to prevent the worst-case scenario, even if there is no appropriate indication [45]. Additionally, women, families and health providers might perceive more intervention and more technology to be the best possible care. As a result the ‘measure of caring is by doing something active, rather than by being present’ [46].

In this study we observed that some interventions, such as IV infusion, catheterization or episiotomies, were provided without proper assessments of clinical signs and symptoms to justify their action. Selective implementation of guidelines and assuming a ‘worst-case’ scenario was also observed for the third stage of labour [7]. Similar to other studies we observed that assessment of completeness of the placenta and regular monitoring the tonus of the uterus was often neglected [28, 47–49]. Instead actions were taken which would normally only be indicated in case of hemorrhage. The apparent routine use of uterine massage and manual expression to empty the uterus for blood clots not only causes unnecessary discomfort for women but it can also lead to iatrogenic occurrence of post-partum hemorrhage and infection [28]. When AMTSL is primarily implemented as a method to prevent post-partum hemorrhage, activities that function to control the natural processes through quality routine assessment and monitoring might seem less important.

### Moving beyond saving lives

Our findings can be seen as examples of a how a global technocratic approach to maternity care, with implementation of policies, systems and interventions focused on prevention and treatment of complications, has disproportionally influenced local practice. As a result there is a large implementation gap for routine management of childbirth [49]. Both the partograph and AMTSL have been implemented primarily with the aim to identify and detect pathology (e.g prolonged labour and post partum haemorrhage) and have also been evaluated as such [21, 50]. This selective focus on some components of interventions indirectly influences care provision as health providers prioritize those same few activities, they feel they will be held accountable for [46]. Global maternal health actors have paid much less attention to their function for controlling the physiology of the birthing process [4, 51]. Guidelines and indicators are focused on those care activities, for which there is sufficient evidence it improves outcomes, not if they improve the process and quality of care. For example, the WHO recommendation for PPH presents that the most important AMTSL component is the administration of a uterotonic [50]. While this is arguably true for the outcome of hemorrhage, our examples show that leaving out some other essential components of AMTSL can have negative consequences.

When global clinical management tools such as the partograph are implemented without locally adapted guidance on how to use such tools within the given context, health providers might resort to alternative interventions or avoid their use all together. In order to motivate health providers for routine birth monitoring, guidelines should be developed which take into account the contextual challenges, within a range of what can be considered acceptable care rather than based on an unrealistic ideal situation. Development of locally achievable guidelines for partograph use have been demonstrated successfully in a recent study in Zanzibar [52, 53]. Continuous advocacy for availability of EmOC remains important, however strict implementation of EmOC does not encompass the physiological and mental processes that are essential to ensure quality care for all women [54, 55]. Efforts to improve intrapartum care need to ensure care is provided respectful to the birthing woman and her newborn [3]. It is increasingly acknowledged that conditions which underlie sub-optimal quality of care enforce the occurrence of abuse and disrespect in childbirth [56]. Women’s denial of quality childbirth care which at the same time increases their vulnerability to abuse are violations of women’s basic human rights [57, 58]. Increasing quality of routine care should start with enabling health providers to return to the basics of what is required to care for women during childbirth within their local context. We cannot expect health providers to timely identify and tackle complications if they are unable to recognize what constitutes a normal birthing process. Nor if their working conditions, including lack of resources limit their possibilities for providing quality care even further*.*

Maternity care in low-resource settings needs to be ‘re-humanized’ ensuring the dignity and the worthiness of the birthing woman and her family are placed at the centre [59]. This requires attention beyond our currently prioritized indicators, where maternity care is restructured and reorganized not based on an industrial model, but on a more humanistic model, making use of the rich evidence-base from midwifery science [19, 45, 55]. There are numerous interventions that have shown to be effective both to improve quality and outcomes of care. Continuous and caring support during birth, the presence of a birth companion, encouraging free movement, and having the birth position of choice [60, 61] are examples of low-cost evidence based interventions that have long been recommended [26] but are neglected. It can be expected that such interventions increase timely access to services, reduce the number of unnecessary interventions and increases satisfaction of care. Ultimately such improvement in the quality of routine care will likely lead to a reduction of unnecessary maternal and perinatal morbidity and mortality.

## Conclusion

In settings with persisting high maternal and perinatal mortality and morbidity, interventions to tackle complications and emergencies seem to have priority over routine care provision. However, the majority of women will likely give birth without the need for interventions. The absence of quality routine care puts many women unnecessarily at risk. Health providers are experiencing pressure of responsibility for outcomes of care processes they perceive to be are out of their control. As a result both underuse and overuse of interventions result in poor quality of routine care for women having uncomplicated births. Insufficient monitoring leads to poor preparedness of health providers both for uncomplicated births and in case complications arise. If we truly want to improve the quality of care for all women attention needs to be paid to the implementation gaps that exist. Complex evaluations that embrace the intricacies of maternity care in low resource settings are needed to identify and understand these gaps. Interventions we know work, can only be effective if they are implemented within a health system that is responsive, encourages contextual adaptation and takes care of placing women’s dignity at its centre.

## Additional files

Additional file 1: Checklist L&D observation tool. (DOCX 42 kb)

Additional file 2: Example analysis process. (XLSX 1574 kb)

## Abbreviations

AMTSL: Active Management of Third Stage of Labour
Bpm: Beats per minutes
EmOC: Emergency obstetric care
MMR: Maternal mortality ratio
PPH: Post partum hemorrhage
WHO: World health organization

## Footnotes

1 We refer to childbirth as ‘labour’ only in reference to stages of labour as this is the common way to describe the stages. In all other cases we speak of ‘birthing’ or the ‘birth process’.

2 During the study period there were several news items describing how health workers were fired after families had demanded someone to be held accountable for the death of their newborns, health workers would share such stories and were afraid to document their names in patient files out of fear that it could be used in court.

3 The co-authors have observed around 50-60 maternities all over SSA over a long time period.
