# Supplementary material for: Quality of care during childbirth in Tanzania: identification of areas that need improvement
Source: Reprod Health. 2018 Jan 27;15:14. doi: 10.1186/s12978-018-0463-1 (PMC5787311; doi:10.1186/s12978-018-0463-1)
Supplement: Supplementary file 1 — Checklist L&D observation tool. (DOCX 42 kb) [file 12978_2018_463_MOESM1_ESM.docx]

| **Data collection tool: Checklist L&D observation** | |
| --- | --- |
| **Category** | **Observation** |
| **1. Basic info** | 1. Date of observation: _____ 2. Name of data collector: ______ 3. Name of facility: ______ 4. HCW ID: ____ 5. Ask consent from health worker (Y/N) and pregnant woman (Y/N)   *Before observing labour, make sure to obtain permission from both the service provider and the client. Also make sure that the provider knows that you are not there to evaluate him or her and that you are not an ‘expert’ to be consulted during the session.* |
| **2. Basic labour info** | 1. Time of arrival of client: _____ 2. Time of being attended by HCW: ____ 3. Client is eligible for observation: Y/N 4. Where is client coming from: Other health facility, ANC ward/OPD, Home |
|  | |
| **3. Introduction and history taking** | 1. Location of introduction/history taking:______ 2. Asks for the following details    - Age (Y/N)    - No of pregnancies (Y/N)    - No of deliveries (Y/N)    - No of live births (Y/N)    - Length of pregnancy (gestational age (Y/N)    - Checks HIV status Y/N 3. Asks if woman has experienced any of the following for current pregnancy:    - Vaginal bleeding (Y/N)    - Fever (Y/N)    - Severe headache and/or blurred vision (Y/N)    - Swollen hands, face, feet (Y/N)    - Convulsions or loss of consciousness (Y/N)    - Severe difficulty breathing (Y/N)    - Persistent couch for 2 weeks or longer (Y/N)    - Severe abdominal pain (Y/N)    - Whether he client has felt a decrease or stop in fetal movement (Y/N)    - If there are any other problems the client is concerned about (Y/N)    - Not asked (Y/N) 4. Client has previous pregnancies (Y/N), *if Yes continue, if No go to no 14* 5. Asks about complications during previous pregnancies and deliveries:    - Heavy bleeding during or after delivery (Y/N)    - Anemia (Y/N)    - High blood pressure (Y/N)    - Convulsions (Y/N)    - Multiple pregnancies (Y/N)    - Prolonged labor (Y/N)    - C-section (Y/N)    - Assisted delivery (Y/N)    - Prior neonatal death (death of baby less than 1 month old) (Y/N)    - Prior stillbirth (baby born dead that does not breathe or cry (Y/N)    - Prior abortion (loss of pregnancy) (Y/N)    - Not asked (Y/N)    - If neonatal death/stillbirth/abortion asks about months of pregnancy Y/N/DK 6. Documents findings:    - On ANC card (Y/N)    - On plane paper (Y/N)    - Other (Y/N)    - No documentation (Y/N) |
| **4. Examination** | 1. Location of examination________ 2. Performs the following activities:    - Washes hands with soap and water or uses alcohol before any initial examination (Y/N)    - Takes temperature (Y/N)    - Takes pulse (Y/N)    - Takes BP (Y/N)    - Asks about condition of urine or checks it (dipstick) (Y/N)    - Checks for signs of anemia (Y/N)    - Checks for signs of oedema (Y/N)    - Checks fundal height (Y/N)    - Checks fetal presentation by palpation of abdomen (Y/N)    - Checks fetal heart rate (Y/N)    - Checks frequency of contractions (Y/N)    - Performs vaginal examination (Y/N) 3. Stage of labour    - Active labor (Y/N)    - Latent labor (Y/N)    - Unknown [if findings are not clear to the observer] (Y/N) 4. Action taken after examination/initial assessment:    - Is referred to non-active stage room (if applicable) (Y/N)    - Is referred to labour room (if applicable) (Y/N)    - Is referred home and told to come back later (Y/N)    - Remains on current location (Y/N) 5. Documents findings    - On ANC card (Y/N)    - On plain paper (Y/N)    - On partogram (Y/N)    - No documentation (Y/N) |
| **ANC card documentation**  *Observer: check ANC card for information (If No ANC card, skip)* | 1. Age_____ 2. Pregnancy____ 3. Parity____ 4. Live births____ 5. LNMP_____, 6. EDD____ 7. Complications during previous pregnancy/childbirth    - Heavy bleeding during or after delivery (Y/N)    - Anemia (Y/N)    - High blood pressure (Y/N)    - Convulsions (Y/N)    - Multiple pregnancies (Y/N)    - Prolonged labor (Y/N)    - C-section (Y/N)    - Assisted delivery (Y/N)    - Prior neonatal death (death of baby less than 1 month old) (Y/N)    - Prior stillbirth (baby born dead that does not breathe or cry) (Y/N)    - Prior abortion (loss of pregnancy) (Y/N) 8. Identifies if high risk Y/N/DK *(Yes if mentions it or documents it, No if not identified but is present, DK if unknown to the observer).* |
|  | |
| **5. Progress of labour** | **Monitoring and detection of abnormal findings on paper documents** |
| **6.Preparation of Labour** | 1. Location of preparing for labour 2. Preparation of essential equipment    - Oxytocin (Y/N)    - Self-inflating ventilation bag (Y/N)    - Newborn mask (Y/N)    - Suction bulb (Y/N)    - Catheter (Y/N)    - Cord-ties (Y/N)    - Delivery pack (Y/N)    - Gloves (Y/N)    - IV cannula’s (Y/N)    - IV fluids (Y/N) |
| **7. Delivery care** | 1. Location of delivery care________ 2. Performs the following actions:  - As baby’s head is delivered supports perineum Y/N - Waits for contraction for delivery of the shoulders if this does not come spontaneous Y/N - Time of birth is checked and mentioned by HCW Y/N  1. Record time of birth:_______ |
| **8. Immediate care** | *Record whether provider carried out the following steps and/or examinations (some of the steps may be preformed simultaneously or by more than one provider)*   1. Checks for another baby **(**Y/N) [*If second baby then stop observation]* 2. Record time uterotonics given___ *(999 if not given)* 3. Which uterotonic given  - Oxytocon - Ergometrine - Syntometrine - Misoprostol  1. Record timing of cord-clamp____  - Applies traction to the cord while applying suprapubic counter pressure Y/N - Slowly delivers the placenta and membranes Y/N - Performs uterine massage after delivery of the placenta Y/N - If uterotonics were given was placenta delivered before uterotonics? (Y/N) - Assesses completeness of placenta and membranes Y/N/DK (*If not complete stop observation)* - Performs manual removal of clots Y/N  1. Tears  - Assesses for perineal and vaginal lacerations Y/N - Repairs tears Y/N - If repair: uses anesthesia for repair Y/N  1. Neonatal care (golden minute)  - Immediately places baby skin to skin Y/N - In case of meconium applies suction prior to stimulation Y/N - Immediately dries baby and stimulates Y/N - Is the baby crying? Y/N (if yes continue to 37)  1. Resuscitation: Performs following procedures when needed  - Dries the baby/stimulation Y/N - Clears the airway/stimulation Y/N - Cuts the cord and brings to table Y/N - Performs inflation breaths Y/N - Calls for help Y/N - Improves ventilation Y/N - Checks for heart rate Y/N - Performs heart massage Y/N  1. Documentation  - Documents delivery process on ANC card Y/N - Documents delivery process in notes and government books Y/N |
| **9. Outcome** | 1. Neonatal condition after birth: Alive (to 43) /death (to 44) 2. If Alive: Condition; good, fair, not-good 3. If Death: Macerated stillbirth, fresh stillbirth, neonatal death 4. Maternal condition after birth: Alive (to 46) /death (to 47 ) 5. If Alive: Condition: good/fair/not-good (go to 44) 6. If Death: Cause of death:_______ 7. If Condition not good why:________ |

| **Patient ID:** | | **Monitoring sheet for L&D observation** | | | | | | | **HCW ID:** |
| --- | --- | --- | --- | --- | --- | --- | --- | --- | --- |
| **Admission time:** | | **Admission Cx:** | | **Cx full time:** | | **Head visible/Pushing time:** | | | **Delivery time:** |
| **Action** | **Time** | **Time** | **Time** | **Time** | **Time** | **Time** | **Time** | **Time** | **Comment** |
| **Checks**  **BP** |  |  |  |  |  |  |  |  |  |
| **Listens to**  **FHR** |  |  |  |  |  |  |  |  |  |
| **Performs**  **VE** |  |  |  |  |  |  |  |  |  |
| **Checks for contractions** |  |  |  |  |  |  |  |  |  |
| **Fills in or reviews**  **partogram** |  |  |  |  |  |  |  |  |  |
| **Other (specify)** |  |  |  |  |  |  |  |  |  |
| **Abnormal findings** (As communicated by nurse, documented or observed by observer)  (H=help, S=supervisor, AROM=artificial rupture of membranes, C=catheterization, Ep=episiotomy, FE=fundal expression, Me=medication, Mo=monitoring, N=none, O=other, R=referral, V=vacuum | | | | | | | | | |
| **Finding** | **Time** | **Action** | **Time** | **Action** | **Time** | **Action** | **Time** | **Action** | **Comment** |
| **FHR problem** |  |  |  |  |  |  |  |  |  |
| **Bleeding** |  |  |  |  |  |  |  |  |  |
| **Meconium** |  |  |  |  |  |  |  |  |  |
| **High BP** |  |  |  |  |  |  |  |  |  |
| **Fever** |  |  |  |  |  |  |  |  |  |
| **Malpresentation** |  |  |  |  |  |  |  |  |  |
| **Poor progress** |  |  |  |  |  |  |  |  |  |
| **Other (specify)** |  |  |  |  |  |  |  |  |  |
| **Unnecessary intervention or harmful practice**  Episiotomy without indication, enema, pubic shaving, automatic intravenous preparation or fluids, manual clots removal after labour, applying fundal expression | | | | | | | | | |
| **Action (specify)** | **Description** | | | | | | | | |
|  |  | | | | | | | | |
|  |  | | | | | | | | |
|  |  | | | | | | | | |
